# Sigmoid colonic tuberculosis presenting as a colovesical fistula mimicking colorectal malignancy: a case report

**DOI:** 10.3389/fmed.2026.1857599

**Published:** 2026-06-08

**Authors:** Lingfeng Zhong, Kun Xia, Yanyun Fan

**Affiliations:** 1 Department of Gastroenterology, The National Key Clinical Specialty, Zhongshan Hospital of Xiamen University, School of Medicine, Xiamen University, Xiamen, Fujian, China; 2 Xiamen Key Laboratory of Intestinal Microbiome and Human Health, Zhongshan Hospital of Xiamen University, School of Medicine, Xiamen University, Xiamen, Fujian, China; 3 Xiamen Gastroenterology Quality Control Center School of Medicine, Xiamen, Fujian, China

**Keywords:** colorectal neoplasms, colovesical fistula, Crohn's disease, intestinal tuberculosis, sigmoid colon

## Abstract

**Background:**

Intestinal tuberculosis (ITB) most commonly involves the ileocecal region. Isolated sigmoid colonic tuberculosis complicated by a colovesical fistula is extremely rare and may closely mimic colorectal malignancy or Crohn's disease (CD).

**Case presentation:**

A 73-year-old man presented with subacute diarrhea, fever, and lower urinary tract symptoms. Laboratory tests showed markedly elevated inflammatory markers and anemia. Cross-sectional imaging demonstrated segmental thickening of the sigmoid colon, pericolic lymphadenopathy, multiple serous effusions, and findings consistent with a colovesical fistula, including bladder wall disruption and intravesical gas. Colonoscopy revealed a circumferential stenosing lesion with irregular ulceration, raising strong suspicion for colorectal malignancy or CD.

**Diagnostic assessment and intervention:**

Initial histopathology showed only mixed inflammatory cell infiltration without granulomas or malignant cells, and empirical antimicrobial therapy failed to control the fever. Given the positive immunological testing for tuberculosis and persistent clinical suspicion, acid-fast bacilli staining and metagenomic next-generation sequencing (mNGS) were performed on colonic biopsy tissue. Acid-fast bacilli were detected, and mNGS identified Mycobacterium tuberculosis complex, confirming ITB. Standard anti-tuberculosis therapy was initiated, leading to rapid clinical improvement, complete endoscopic mucosal healing, and radiological resolution of the colovesical fistula.

**Conclusion:**

This case highlights that ITB can present as an isolated tumor-like sigmoid lesion complicated by fistula formation. When routine histology is nondiagnostic, especially in the absence of granulomas, integration of imaging, immunological testing, special staining, and molecular diagnostics may be crucial for early diagnosis, avoidance of misdiagnosis, and timely targeted treatment.

## 1 Introduction

Intestinal tuberculosis (ITB) remains an important clinical entity in the context of the global resurgence of tuberculosis. It most commonly involves the ileocecal region and

typically presents with hypertrophic or ulcerative lesions. In contrast, isolated involvement of the sigmoid colon is extremely rare and may be under-recognized by clinicians. Because the sigmoid colon is also a common site for colorectal cancer and Crohn's disease, segmental thickening, circumferential narrowing, and irregular ulceration in this region can easily be misinterpreted as malignancy or inflammatory bowel disease. Colovesical fistula is an uncommon complication of ITB, and an isolated tuberculous fistula arising from the sigmoid colon is even rarer, making the radiologic and endoscopic findings difficult to distinguish from locally advanced colorectal cancer or complicated Crohn's disease.

Reports of ITB presenting as a tumor-like sigmoid lesion with colovesical fistula remain scarce, and most reported cases have relied on typical histological findings, such as caseating granulomas, or mycobacterial culture for diagnosis. The key diagnostic challenge in the present case was that initial histopathology showed only nonspecific inflammation without granulomas, while empirical antimicrobial therapy failed to control the fever. This case therefore highlights an important diagnostic gap: ITB at an atypical site and without characteristic histology may be delayed or misdiagnosed. By presenting this case, we aim to emphasize the need to include ITB in the differential diagnosis of sigmoid tumor-like lesions with fistula formation and to illustrate the value of integrating imaging, special staining, and metagenomic next-generation sequencing (mNGS) for early pathogen confirmation and timely targeted treatment.

## 2 Case presentation and patient information

A 73-year-old man was admitted to our Department of Gastroenterology with a 3-week history of diarrhea and a 1-week history of fever. His medical history included hypertension for 10 years and lacunar cerebral infarction for 2 years, for which he regularly took atorvastatin 20 mg/day and clopidogrel 75 mg/day. He had no known history of tuberculosis, tuberculosis exposure, diabetes, or immunosuppression, and had not received corticosteroids or other immunosuppressive agents. He did not smoke or drink alcohol and had not undergone colonoscopy within the previous 5 years or tuberculosis screening within the previous 2 years.

The illness began with yellow watery diarrhea 7–8 times per day, without fever, hematochezia, or mucus in the stool. During the 2nd week, diarrhea persisted at 5–6 episodes per day, without obvious weight loss. From day 14, he developed afternoon-predominant fever up to 39.0°C, accompanied by urinary frequency, urgency, and dysuria, but without night sweats, cough, nausea, vomiting, or lower limb edema. On day 19, he presented to the emergency department, where inflammatory marker elevation and marked pyuria were noted. Empirical ceftriaxone was administered for 2 days without clinical improvement. He was therefore admitted to our department on day 21 for further evaluation.

**Figure 1:**
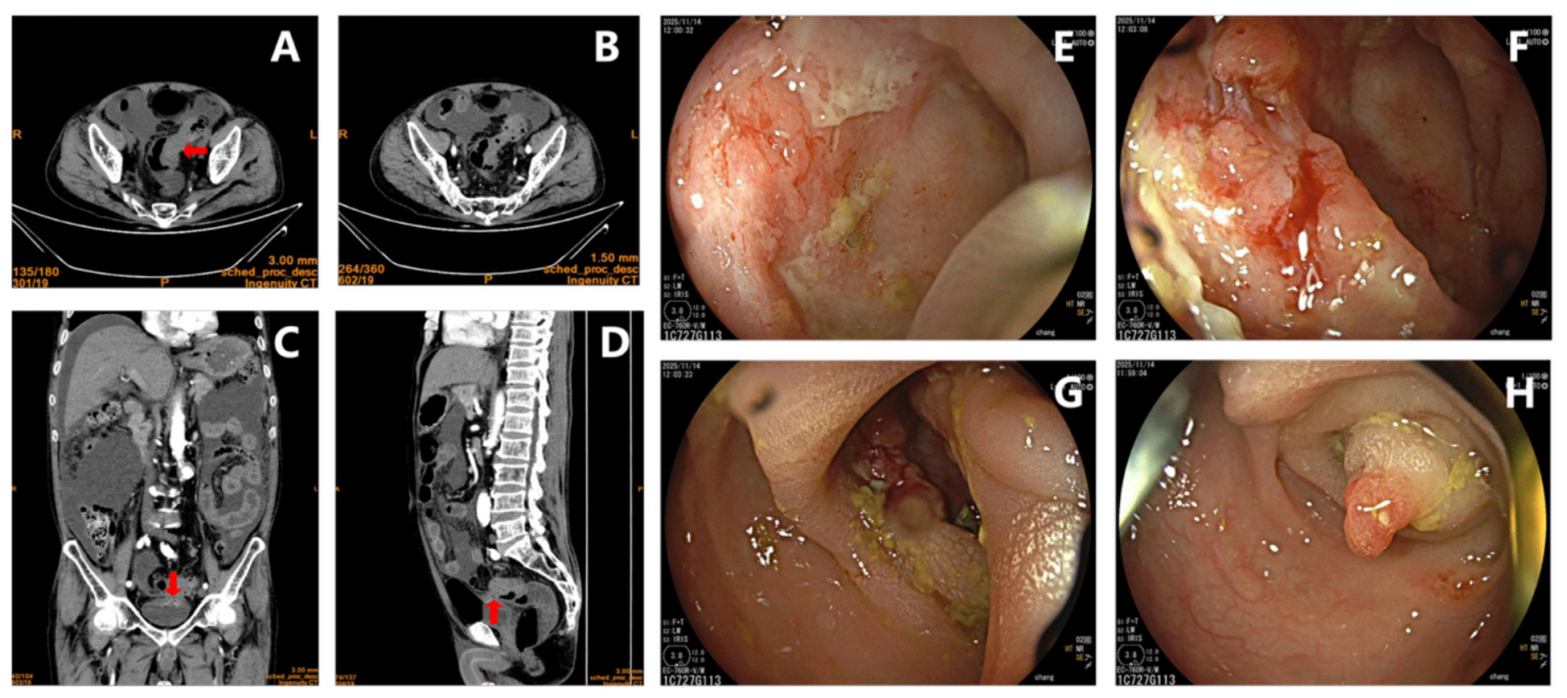
CT and lower gastrointestinal endoscopy findings. **(A, B)** Axial CT image showing sigmoid colonic wall thickening with multiple pericolic lymph nodes. **(C, D)** Coronal and sagittal CT images showing thickening and ulcerative disruption of the posterior bladder wall, with an intravesical air-fluid level. **(E, F)** Endoscopic images showing irregular circumferential ulceration with surface disruption and adherent whitish exudate. **(G, H)** Endoscopic images at 15 cm from the anal verge showing a circumferential mass-like lesion with luminal stenosis, through which the endoscope could not pass. Red arrows indicate the key CT abnormalities.

**Figure 2:**
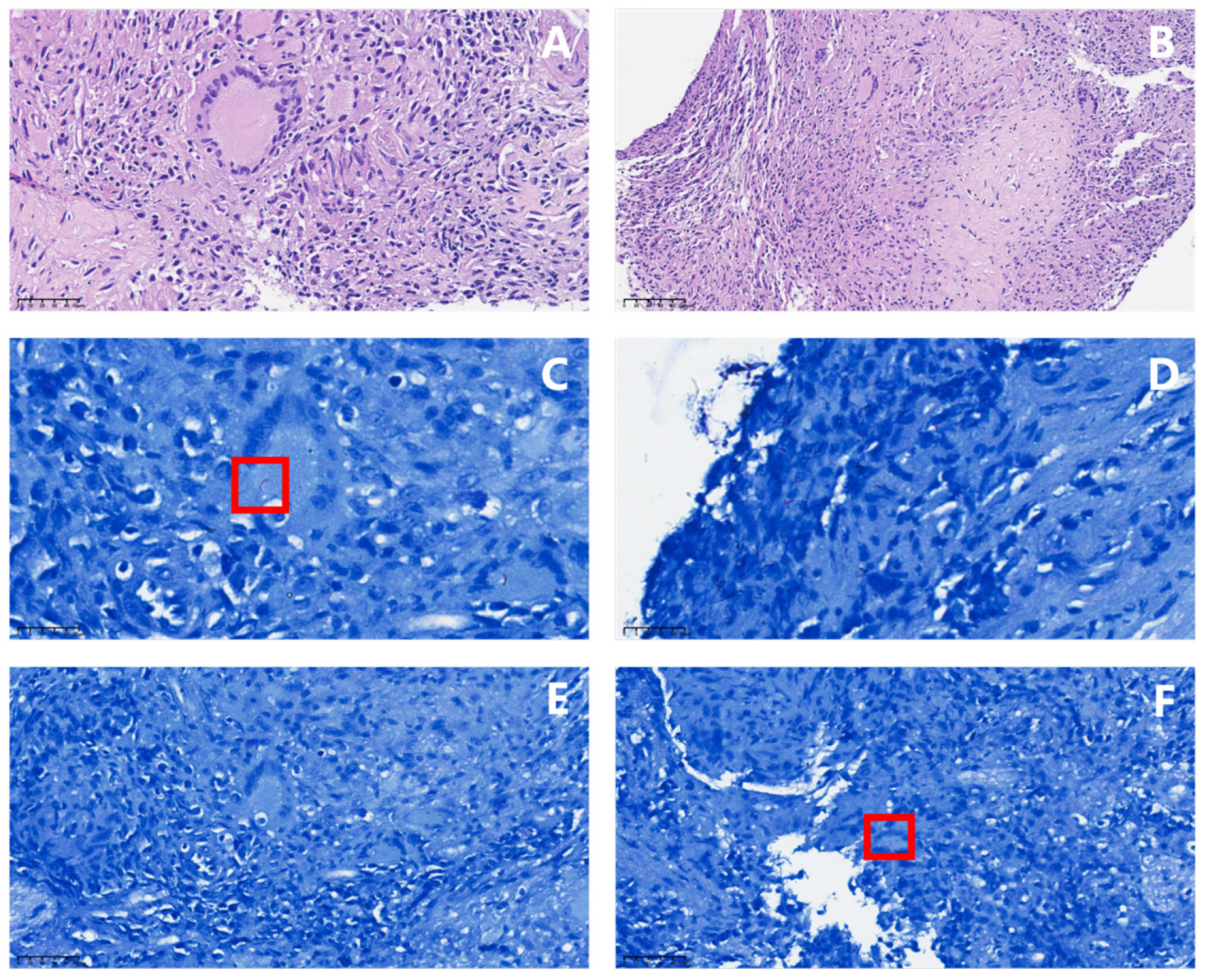
Histopathological findings. **(A, B)** Hematoxylin and eosin-stained sections at 200 × and 100 × magnification, respectively. **(C, D)** Acid-fast-stained sections at 400 × magnification. **(E, F)** Acid-fast-stained sections at 200 × magnification. Red boxes indicate acid-fast bacilli consistent with Mycobacterium tuberculosis.

On admission, physical examination revealed tenderness in the right lower abdomen, mild abdominal guarding, and bowel sounds at 6/min. No oral or genital ulcers were observed. There was no rash or erythema on the extremities, and no perianal fistula or abscess was present. Laboratory tests showed a white blood cell count of 4.62 × 10^9^/L, neutrophils 75.7%, hemoglobin 84 g/L, platelet count 298 × 10^9^/L, D-dimer 3.56 mg/L, C-reactive protein 115.41 mg/L, erythrocyte sedimentation rate 134.70 mm/h, and albumin 29.0 g/L. Stool occult blood was positive. Urinalysis showed protein 1+, hematuria 3+, and leukocytes >100/HPF. T-SPOT.TB was positive and the purified protein derivative test was positive. Coagulation function, renal function, thyroid function, gastrointestinal tumor markers, Epstein–Barr virus, and cytomegalovirus tests were within normal limits. Autoimmune markers and stool tests for common infectious pathogens were negative. Electrocardiography and chest CT showed no significant abnormalities. Abdominal CT and lower gastrointestinal endoscopy findings are shown in Figure 1. Biopsies were obtained from the sigmoid colonic lesion, and histopathological findings are shown in Figure 2.

## 3 Diagnostic assessment

The patient was a 73-year-old man with a subacute course of diarrhea, fever, and lower urinary tract symptoms, accompanied by markedly elevated inflammatory markers and anemia. Abdominal CT showed mild segmental wall thickening of the sigmoid colon, pericolic lymphadenopathy, multiple serous effusions, blurred pericolic fat planes, and findings suggestive of a colovesical fistula, including posterior bladder wall disruption and intravesical gas. Colonoscopy revealed a circumferential mass-like stenosing lesion in the sigmoid colon with irregular ulceration, without an identifiable fistula opening. Initial biopsy showed mixed inflammatory cell infiltration, including lymphocytes, plasma cells, neutrophils, and fewer than 10 eosinophils per high-power field, but no granulomas, malignant cells, or viral inclusions.

Based on these findings, the main differential diagnoses included colorectal cancer, intestinal lymphoma, CD, and, less commonly, ITB. Colorectal cancer was considered less likely because serum tumor markers, including CEA and CA19-9, were within normal ranges, CT showed no liver or distant metastasis, and and 6–8 deep biopsy samples showed no dysplasia or adenocarcinoma. Intestinal lymphoma was also considered unlikely because there was no generalized lymphadenopathy, peripheral blood smear and flow cytometry showed no clonal lymphocyte population, and biopsy immunohistochemistry demonstrated a mixed polyclonal inflammatory pattern without light-chain restriction.

CD was an important differential diagnosis because it may present with segmental, penetrating disease and fistula formation. However, several features were atypical for CD: there was no perianal fistula, abscess, or skin tag; colonoscopy did not show longitudinal ulcers, cobblestone appearance, or aphthous ulcers; biopsy showed no non-caseating granulomas, basal plasmacytosis, or neural hyperplasia; and the lesion was confined to the sigmoid colon without typical ileocecal involvement or skip lesions. Therefore, CD was considered less likely.

Given the known difficulty in differentiating ITB from CD, we also considered an algorithmic diagnostic approach. Hilmi et al. proposed a practical algorithm integrating clinical features, ileocolonoscopy with biopsies, tuberculosis work-up, and reassessment to distinguish CD from ITB (1). In our patient, the absence of typical CD features, positive tuberculosis-related tests, and subsequent microbiological and molecular evidence supported ITB.

Despite the absence of granulomas on routine histology, ITB remained a strong consideration. The supporting features included subacute fever, multiple serous effusions, colovesical fistula formation, a positive T-SPOT.TB result, a positive purified protein derivative test with a 15-mm induration, and persistent fever despite 5 days of empirical broad-spectrum antimicrobial therapy with ceftriaxone and metronidazole. Therefore, additional microbiological and molecular tests were pursued.

On day 8 after admission, acid-fast bacilli staining was requested using the original paraffin-embedded biopsy tissue, and mNGS was performed on the colonic biopsy specimen. On day 10 after admission, acid-fast staining revealed several acid-fast bacilli. Because the bacillary load was low and species-level confirmation was required, mNGS was used for pathogen identification. mNGS identified Mycobacterium tuberculosis complex, with 63,073 specific sequence reads and a relative abundance of 92%, while no other relevant pathogens, including non-tuberculous mycobacteria, Nocardia, or fungi, were detected. Mycobacterial culture was not performed because of its long turnaround time, and conventional PCR was not performed because of local workflow constraints.

Based on these findings, the final diagnosis was isolated sigmoid colonic tuberculosis complicated by a colovesical fistula (Figures 2C–F).

## 4 Therapeutic intervention and follow-up

After acid-fast bacilli staining and mNGS supported the diagnosis of ITB, infectious disease specialists were consulted, and standard anti-tuberculosis therapy was initiated. During the intensive phase, the patient received oral rifampicin 0.6 g once daily, isoniazid 0.3 g once daily, ethambutol 1.0 g once daily, and pyrazinamide 0.5 g twice daily. The patient weighed 68 kg. The pyrazinamide dose was reduced because of mildly elevated baseline serum uric acid (486 μmol/L), after consultation with the infectious disease team.

On day 10 of treatment, the patient developed mild nausea and decreased appetite, while liver function and bilirubin levels remained normal. To improve tolerability, pyrazinamide was changed to postprandial administration, and vitamin B6 25 mg/day was added to prevent isoniazid-related neurotoxicity. The symptoms resolved within 1 week, and no further treatment modification was required. No rash, visual disturbance, arthralgia, or severe adverse event occurred. Liver function, renal function, serum uric acid, and visual symptoms were monitored monthly.

After 2 months of intensive therapy, fever and diarrhea had resolved, and inflammatory markers had decreased substantially. Ethambutol and pyrazinamide were discontinued, and rifampicin 0.6 g/day plus isoniazid 0.3 g/day were continued for a planned total treatment duration of 6 months. During hospitalization, adherence was ensured by directly observed therapy. After discharge, adherence was assessed by pill counts and a medication diary, with regular outpatient follow-up.

The clinical and radiological response was favorable. At baseline, the patient had daily fever up to 39.0°C, diarrhea 7–8 times per day, CRP 115.41 mg/L, CT evidence of a colovesical fistula, and colonoscopic evidence of circumferential sigmoid stenosis with irregular ulceration. After 2 weeks of treatment, body temperature decreased to below 37.5°C, diarrhea improved to 1–2 times per day, and CRP decreased to 32 mg/L. At 1 month, symptoms had completely resolved, CRP decreased to 8 mg/L, and ESR decreased to 22 mm/h. Abdominal radiography showed disappearance of intravesical gas.

**Figure 3:**
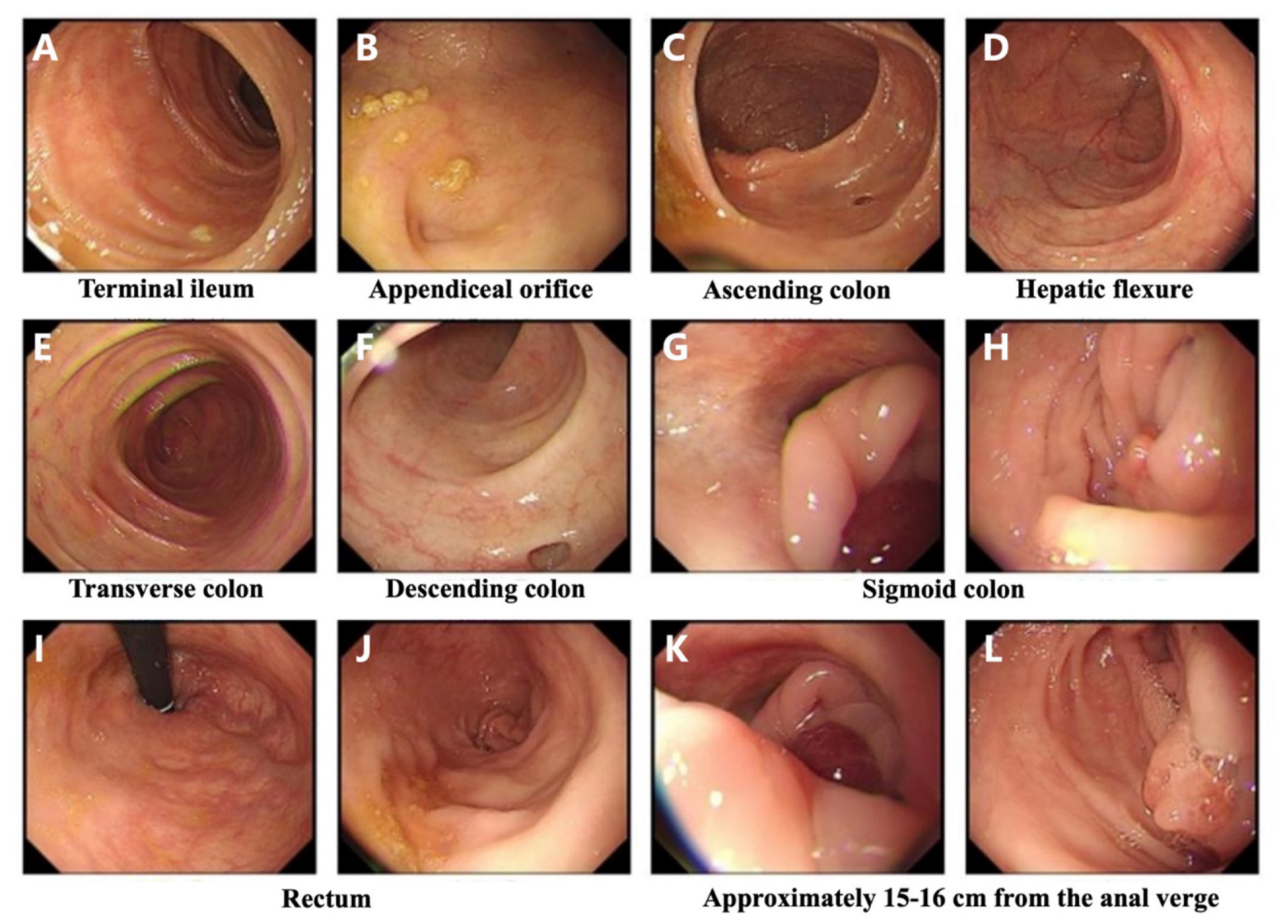
Follow-up colonoscopy after anti-tuberculosis therapy. **(A–J)** Representative endoscopic images of the examined intestinal segments, including the terminal ileum, appendiceal orifice, ascending colon, hepatic flexure, transverse colon, descending colon, sigmoid colon, and rectum. **(K, L)** Follow-up views of the previous sigmoid lesion site, approximately 15–16 cm from the anal verge, showing complete ulcer healing with mild residual mucosal congestion and scar-like changes.

At 2 months, repeat colonoscopy showed marked improvement of the previous sigmoid stenosis, complete healing of the ulceration, and only mild mucosal congestion with scar-like change (Figure 3). Biopsy showed chronic nonspecific inflammation, and acid-fast staining was negative. Repeat CT showed resolution of the sigmoid wall thickening, normalization of pericolic lymph nodes, and complete closure of the colovesical fistula, with restoration of bladder wall continuity and no intravesical gas. At 4 months, telephone follow-up showed no evidence of relapse, and the patient had gained 5 kg. At the end of 6 months of therapy, repeat CT showed no fistula recurrence and normal morphology of the colon and bladder. The patient declined repeat colonoscopy at that time. No recurrence of tuberculosis has been observed during follow-up.

## 5 Discussion

ITB is a chronic inflammatory disease of the intestine caused by Mycobacterium tuberculosis infection. Clinically, it may present with nonspecific symptoms such as abdominal pain, diarrhea, fever, and weight loss. ITB most commonly involves the ileocecal region, where prolonged retention of intestinal contents and abundant lymphoid tissue provide a favorable basis for bacillary colonization. Isolated colonic tuberculosis accounts for an extremely low proportion of abdominal tuberculosis, estimated at only 2%−3%, and represents an even smaller proportion of all gastrointestinal tuberculosis. Therefore, when a lesion is confined to the sigmoid colon, more common diseases such as colorectal cancer, CD, or lymphoma are usually considered first rather than ITB. The present case reflects this rare scenario of isolated sigmoid involvement and demonstrates the ability of ITB to masquerade as a “great mimicker”.

The diagnostic complexity in this case was further increased by colovesical fistula formation. Fistula formation caused by ITB has been reported in the literature. A retrospective analysis of 22 patients with complications of abdominal tuberculosis showed that fistula formation was an important complication among eight patients with intestinal perforation, and the shared radiologic and pathologic features of patients with fistulas included focal or multiple strictures, severe adhesions, and intestinal wall fibrosis (2). However, these complications mostly occur in the ileocecal region or small intestine. Isolated sigmoid involvement complicated by a colovesical fistula is extremely rare in tuberculosis. In our patient, intravesical gas, posterior bladder wall disruption, urinary symptoms, and a stenosing sigmoid lesion initially raised concern for locally advanced colorectal cancer or penetrating CD. The recurrent fever and lack of response to routine antimicrobial treatment further complicated the early clinical judgment.

The central diagnostic dilemma was that multiple biopsies obtained during the initial colonoscopy from the sigmoid stenotic and ulcerative lesion showed only mixed inflammatory cell infiltration, without caseating granulomas or malignant cells. This situation is not uncommon in ITB. A systematic review and meta-analysis showed that, using endoscopic tissue biopsy specimens, the pooled sensitivity of acid-fast bacilli detection was only 12% (95% CI, 8%−17%), and the pooled sensitivity of histopathological detection of caseating granulomas was only 18% (95% CI, 12%−27%) (3). Because tuberculous lesions are often located in the submucosa or deeper layers, colonoscopic biopsy may sample only superficial mucosal tissue, leading to false-negative results. Although Mycobacterium tuberculosis culture is regarded as the diagnostic “gold standard,” its sensitivity is also limited. The above-mentioned meta-analysis showed that the pooled sensitivity of liquid culture was only 25% (95% CI, 13%−43%) (3), and culture requires several weeks. The positivity rate of acid-fast staining is likewise limited, with a sensitivity of only 17.3%−31.0% (4). Thus, negative routine histology should not exclude ITB when clinical suspicion remains high.

Given these limitations, integration of multiple diagnostic methods is essential for improving the diagnostic accuracy of ITB. Kudu and Daniş noted that the diagnostic challenge of gastrointestinal tuberculosis arises from nonspecific symptoms and the absence of a highly accurate diagnostic algorithm, and that diagnosis requires integration of clinical suspicion, imaging, endoscopy, histopathology, microbiology, and molecular methods (5). Interferon-gamma release assays (IGRAs) can serve as screening and supportive diagnostic tools rather than confirmatory tests (6). The above-mentioned meta-analysis showed that the pooled sensitivity and specificity of IGRA were 86% (95% CI, 79%−91%) and 86% (95% CI, 81%−89%), respectively, indicating good diagnostic accuracy and potential value when combined with traditional methods to improve detection. In our patient, a positive T-SPOT result was one of the key factors that raised clinical suspicion of ITB, but final pathogen confirmation still depended on pathogen detection.

In this case, the key step was the prompt initiation of special staining and molecular testing despite negative routine pathology. Acid-fast staining provided an important clue, but the final diagnosis relied on mNGS. As an emerging molecular diagnostic technology, mNGS does not depend on culture and can detect microorganisms, including rare pathogens, within a short time. It has unique advantages in the diagnosis of extrapulmonary tuberculosis and can identify pathogens that may not be detected by conventional methods (7, 8). In this patient, mNGS confirmed the presence of Mycobacterium tuberculosis and excluded other pathogens, including non-tuberculous mycobacteria and fungi. Therefore, when clinical suspicion remains high, molecular testing such as mNGS or GeneXpert should be considered early, even if routine histology, smear, or culture is negative.

Several limitations should be acknowledged. First, although mNGS was diagnostically useful, it remains costly and is not yet widely available in some regions of China. In resource-limited settings, diagnosis may still depend on conventional culture, which requires 4–8 weeks, or GeneXpert testing. Second, the diagnosis was based on colonoscopic biopsy rather than a full-thickness surgical specimen. However, because the patient responded rapidly to anti-tuberculosis therapy and imaging showed no clear surgical indication, conservative treatment was preferred. Third, mycobacterial culture and drug-susceptibility testing of intestinal tissue were not obtained; therefore, information on drug resistance was unavailable. Finally, as this is a single case report, the generalizability of its conclusions is limited.

Overall, this case highlights that ITB should remain in the differential diagnosis of atypical segmental colonic lesions, even when the lesion is located in the sigmoid colon and routine histology does not show granulomas. In patients with subacute fever, diarrhea, elevated inflammatory markers, pericolic lymphadenopathy, serous effusions, or fistula formation, clinicians should maintain suspicion for ITB. Early integration of imaging, endoscopy, immunological testing, special staining, and molecular diagnostics may help establish the diagnosis and prevent delayed or inappropriate treatment.

## Data Availability

The original contributions presented in the study are included in the article/supplementary material, further inquiries can be directed to the corresponding authors.
